# Multispectral Benchmark Dataset and Baseline for Forklift Collision Avoidance

**DOI:** 10.3390/s22207953

**Published:** 2022-10-19

**Authors:** Hyeongjun Kim, Taejoo Kim, Won Jo, Jiwon Kim, Jeongmin Shin, Daechan Han, Yujin Hwang, Yukyung Choi

**Affiliations:** Department of Intelligent Mechatronics Engineering, Sejong University, Seoul 05006, Korea

**Keywords:** automated forklifts, intralogistics, collision avoidance, pedestrian detection, multispectral, 2.5D detection

## Abstract

In this paper, multispectral pedestrian detection is mainly discussed, which can contribute to assigning human-aware properties to automated forklifts to prevent accidents, such as collisions, at an early stage. Since there was no multispectral pedestrian detection dataset in an intralogistics domain, we collected a dataset; the dataset employs a method that aligns image pairs with different domains, i.e. RGB and thermal, without the use of a cumbersome device such as a beam splitter, but rather by exploiting the disparity between RGB sensors and camera geometry. In addition, we propose a multispectral pedestrian detector called SSD 2.5D that can not only detect pedestrians but also estimate the distance between an automated forklift and workers. In extensive experiments, the performance of detection and centroid localization is validated with respect to evaluation metrics used in the driving car domain but with distinct categories, such as hazardous zone and warning zone, to make it more applicable to the intralogistics domain.

## 1. Introduction

According to the paper [1], forklifts were the sole cause of 78 work-related fatalities and 7290 non-fatal injuries requiring days away from work in 2020. To prevent accidents caused by humans, automated forklifts play a crucial role in automated logistics. However, automated forklifts cannot prevent accidents entirely; accidents may occur when automated forklifts fail to recognize workers due to factors such as lighting conditions and occluded or truncated workers. Therefore, it is necessary to incorporate human awareness into automated forklifts so that they can operate effectively in the aforementioned challenging environments.

Pedestrian detection is one of the important technologies that can be used to assign a human-aware property to automated forklifts in order to avoid unexpected accidents, such as collisions, at an early stage. Typically, pedestrian detection for automated forklifts can be simply performed with RGB cameras, but RGB cameras are not robust to low illumination, complex background, and irregular lighting conditions, resulting in failure under these conditions. For this reason, research on multispectral pedestrian detection has been conducted in autonomous driving [2,3,4] and visual surveillance [5], utilizing RGB cameras to capture abundant content information and thermal cameras to capture infrared radiation. Utilizing the benefits of each camera, these studies were able to increase discriminability between instances by using rich content information from RGB cameras and could successfully detect pedestrians in a variety of challenging environments by using the environmental robustness of thermal cameras.

Despite the advantages of multispectral pedestrian detection models, it is challenging to implement them in the real-world. For example, in multispectral setups, aligned image pairs are preferred to reduce annotation costs and enhance detection performance; however, it is difficult to align RGB and thermal images due to the dissimilar nature of the two domains. Specifically, a disparity between image coordinates occurs during the process of projecting the same 3D information into different 2D image coordinates. The disparity can be calibrated by matching pixel pairs in parallel regions that are rectified by transforming epipolar lines that lie between the two images. In this case, cost matching between pixel intensities is required, but this cannot be accomplished easily due to the modality gap between the RGB and thermal domains. For this reason, in the KAIST dataset, which is the most well-known benchmark dataset for multispectral pedestrian detection, a beam splitter is used to overlay RGB and thermal images in the same image coordinates to produce a pair of registered images. Although this method was effective at aligning images, it could not be used for practical applications due to the configuration of the sensor, which requires a beam splitter that is too cumbersome and sensitive to small changes and vibrations.

**Figure 1:**
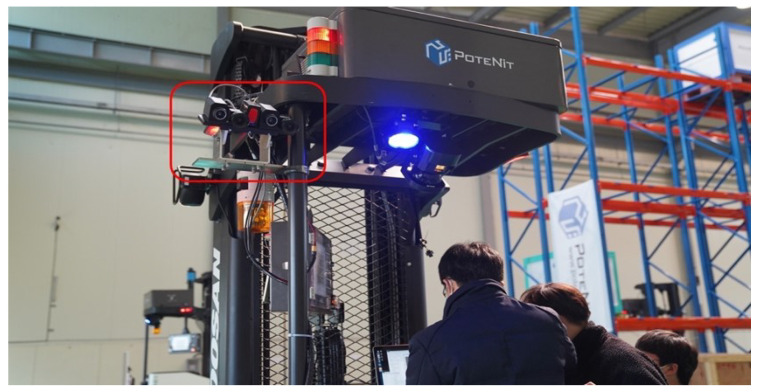
The camera system mounted on the actual automated forklift (Red box).

To this end, in this paper, we present a pixel-level aligned dataset taken with four cameras equipped on a real driving automated forklift, as shown in Figure 1. In the dataset, an image registration technique is used to obtain aligned RGB and thermal image pairs without the use of a cumbersome device by taking advantage of the disparity between RGB-RGB images and camera geometry. Using this method, we acquire a pixel-level aligned multispectral pedestrian dataset for automated forklifts captured with stereo multispectral sensors and a real driving automated forklift for intralogistics. The distances between the camera and pedestrians are then annotated based on the location of the pedestrian in the image and stereo-camera characteristics. Specifically, the dataset comprises 19.4 k frames including 20.2 k pedestrians with occlusion labels depending on how much the pedestrian is occluded as follows: (1) no-occlusion (0%); (2) partially-occluded (0–50%); (3) strongly-occluded (50–100%).

In addition, we propose a 2.5D multispectral pedestrian detector, namely SSD 2.5D, which is capable of detecting pedestrians and simultaneously localizing them in 3D space in order to avoid workers and prevent collisions. The proposed model is based on SSD, a widely used baseline network in the field of multi-spectral pedestrian detection; consequently, it is designed to take multiple inputs, i.e., RGB and thermal images, and combine RGB and thermal features in middle convolution layers. For centroid localization of pedestrians, we added a centroid regression branch to localize the centroid of pedestrians in 3D space, i.e., the distance between a forklift and workers. To validate the proposed model, we employ commonly used evaluation metrics in pedestrian detection, including average precision (AP) and log-average-miss-rate (MR) to evaluate detection performance, and percent error and average localization precision (ALP) to evaluate localization performance. On top of that, we define subcategories, such as hazardous zone and warning zone, based on whether the pedestrian is closer or further than a braking distance, and evaluate the proposed model in relation to the criterion.

### 1.1. Related Works

This paper mainly focuses on multispectral pedestrian detection and related datasets for indoor applications in order to attribute a human-aware characteristic to automated forklifts. Therefore, we will not cover all vision-guided vehicle research, but we will discuss multispectral pedestrian detection datasets, algorithms, and indoor applications. For an overview of vision-guided vehicles, please refer to the survey papers [6,7].

#### 1.1.1. Multispectral Pedestrian Detection Datasets

Hwang et al. [8] proposed the KAIST multispectral dataset, which consists of 95,328 fully overlapping RGB-thermal image pairs that have been optically aligned using a beam splitter. The KAIST dataset has been utilized by many researchers due to the absence of a discrepancy problem. However, the sensor pack used in the dataset is too cumbersome, making practical application of the dataset difficult. In order to mitigate the issue, datasets collected with practical sensor configurations [9,10] have also been utilized; however, they contain misaligned RGB and thermal image pairs and are limited to driving road scenarios. In the meantime, Jia X. et al. introduced the LLVIP dataset [5] for visual surveillance, which consists of 16,836 RGB-thermal image pairs, and all image pairs are precisely aligned in time and space. Despite the fact that the dataset is accurately aligned and contains a sufficient number of image pairs, it is still unsuitable for pedestrian detection on automated forklifts due to the characteristics of automated forklifts. First, automated forklifts typically operate indoors (e.g., warehouses), so datasets that only account for outdoor scenarios are not best suited for pedestrian detection on automated forklifts. Second, datasets for automated forklifts should include various viewpoints because automated forklifts are constantly in motion. However, the LLVIP dataset was only captured outdoors, and the camera within the dataset is stationary, as the dataset is intended for visual surveillance. Martin-Martin et al. [11] made an egocentric robot equipped with several sensors, such as LiDAR, RGB-D, RGB, 360-degree fish-eye cameras, etc., and collected indoor/outdoor datasets on the university campus. Although they utilized various sensors with a real commercial robot and included both indoor and outdoor scenes in the dataset, they did not include a thermal camera, so the dataset cannot be used to train models that must operate in low-light environments. In addition, the aforementioned datasets only employed monocular cameras for each sensor domain, whereas a stereo setup is more advantageous for research purposes. To the best of our knowledge, there are only two datasets [12,13] obtained from stereo setups for both RGB and thermal domains. However, none of these datasets pertain to intralogistics, nor do they disclose datasets.

#### 1.1.2. Multispectral Pedestrian Detection Algorithms

Since the release of the KAIST dataset [8], numerous multispectral pedestrian methods for all-day vision have been proposed. J. Li et al. [14] designed four Faster-RCNN [15] based DNN architectures to combine RGB and thermal features at various stages, and empirically analyzed the results to determine the most effective fusion method. To achieve low latency for real-world applications, Roszyk et al. and Cao et al. [16,17] conducted similar experiments with a different architecture baseline, YOLOv4 [18]. According to the findings of these three studies, Halfway Fusion, which combines RGB and thermal branches in middle convolution layers, yields the best results regardless of the base architecture. On top of this knowledge, many studies have proposed multispectral pedestrian detection algorithms and have worked on how to effectively fuse two modalities [2,3,4]. However, these approaches to multispectral pedestrian detection are limited to outdoor road scenes and have not been verified for indoor applications, despite the fact that majority of automated forklifts operate in indoor warehouses.

#### 1.1.3. Pedestrian Detection Algorithms for Intralogistics

Liu et al. [19] collected a custom dataset and designed DNN architectures for accurate recognition of warehouse surroundings without having to add environmental landmarks. Linder et al. [20] proposed a YOLO-based RGB-D fusion method that calculates the 3D localization of humans for intralogistics in addition to detection results. To be specific, they adopt YOLOv3 as a base architecture and design a model that fuses RGB and RGB-D features in middle convolution layers and additionally outputs the centroid location of pedestrians in RGB-D data. In addition to the architecture, they propose the use of synthetic datasets in training and a depth-aware augmentation, a variant of the zoom-in and zoom-out augmentations used in YOLOv3, and achieve state-of-the-art performance on their intralogistics subset. However, the aforementioned methods do not incorporate thermal images and are therefore significantly affected by illumination changes depending on circumstances.

## 2. Materials and Methods

### 2.1. Proposed Multispectral Pedestrian Dataset

This section will discuss synchronization, stereo camera calibration, image alignment, the annotation procedure, and the characteristics of the presented dataset.

#### 2.1.1. Multi-Modal Camera Configuration

**Figure 2:**
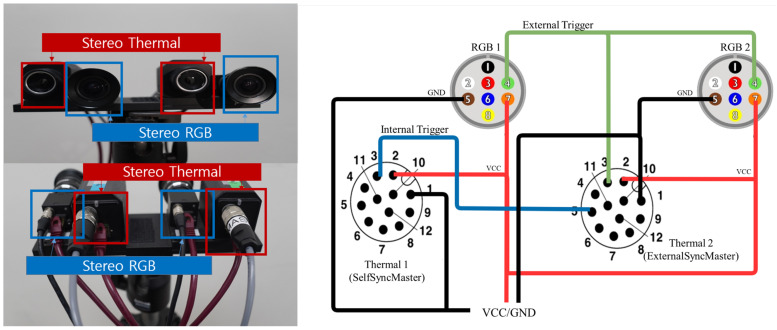
The sensor configuration (**Left**). The system wiring diagram (**Right**), which is used to supply power to the cameras and synchronize them hardware-wise. GND and VCC are represented by the black and red lines, respectively. Synchronization signals originate from Thermal 1 and are transmitted to Thermal 2 via Internal Trigger (Blue line). Then, the Thermal 2 camera distributes synchronization signals via External Trigger to RGB 1 and RGB 2. (Green line).

The proposed sensor system consists of two RGB cameras and two thermal cameras, as depicted in Figure 2, and the gigabit ethernet (GigE) type is used to achieve a fast shutter speed. To collect the dataset, frame-grabbing software was used, such as FlyCapture SDK for RGB and eBus SDK for thermal images, which were provided by the camera manufacturers. The following is a summary of specific hardware information for the proposed sensor system.

- 2 × PointGrey Flea3 color camera (FL3-GE-13S2C 1288 × 964, 1.3 MP, Sony ICX445) GigE 84.9(H) × 68.9(V) with Spacecom HF3.5M-2 Lens 3.5 mm
- 2 × FLIR A35 thermal camera (320 × 256, 7.5 13 μm GigE 63(H) × 50(V) with 7.5 mm.

In most cases, GigE-type cameras are powered by power over ethernet (PoE), which is one type of Ethernet power supply. However, the proposed sensor system leverages general-purpose input-output (GPIO) which is an alternative way to supply power in consideration of lightweight and portability. 14.4V 4C lithium polymer battery is used, and the wiring diagram of GPIO is shown in Figure 2. In order to connect multiple cameras simultaneously to a portable laptop, which has a limited number of ports, an Ethernet-to-USB converter and a USB hub are included in the proposed sensor pack. In addition, the MacBook Pro 2012 Late with USB 2.0 is used to acquire images from four distinct cameras without significant packet loss. Ethernet-to-USB3.0 adapter and USB3.0 hub specifications are summarized as follows.

- j5create USB3.0 Gigabit Ethernet Adapter (JUE130).
- j5create USB3.0 HUB (JUH377).

#### 2.1.2. Multi-Modal Sensor Synchronization

For multiple cameras, images must be synchronized precisely; otherwise, significant performance degradation may occur. In the proposed camera setup, each image from multiple cameras is hardware-wise synchronized to overcome the limitations caused by an accumulated error when using software-based synchronization methods on a timestamp basis. As shown in Figure 2, this is accomplished by connecting four cameras using a master-slave interface. The signal for synchronization originates from one of the thermal cameras, which is referred to as self-master, and is transmitted to the other thermal camera, which is referred to as external-master. The signal is then simultaneously distributed to both RGB cameras by the external-master. As a result, four cameras can share synchronization signals, which contributes to the reduction of synchronization error; this direct method of reducing synchronization error is considered a more accurate method of synchronizing cameras than timestamp-based software methods. Figure 2 and Table 1 illustrate the configuration of the setup and the specifications of each camera, respectively.

#### 2.1.3. Multi-Modal Sensor Calibration

Stereo system calibration must be performed by matching image correspondences in both modalities in order to calibrate multispectral cameras. However, a general checkerboard is not visible in the infrared domain, unlike conventional stereo systems. Therefore, Line-board [21], a copper grid pattern board, was utilized to maintain a high contrast in thermal images, as copper lines are more conductive than background areas. By heating the board for a few seconds with a blow dryer or other heat source, the checkerboard maintains a uniform thermal distribution despite its simplicity.

**Figure 3:**
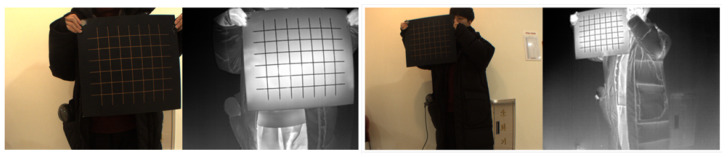
For 0.5m distance, every image is divided into four square-shaped insets to be able to cover all areas on the image when performing a camera calibration (**Left**). Likewise, sixteen insets are used for 1m distance (**Right**).

As shown in Figure 3, all images for the calibration are captured at a distance of 0.5 m from the checkerboard to handle a wider range of lens distortion with fewer images. Due to the differing viewpoints of the cameras, it is difficult to cover all areas of the checkerboard, resulting in an imprecise calibration, especially near the edge lines. To alleviate the issue, we performed additional calibration by adjusting the distance between the checkerboard and camera during the image capturing process.

#### 2.1.4. Multi-Modal Sensor Alignment

Sensor alignment is important in a multispectral setup that uses both RGB and thermal cameras, as it makes information readily available at visually dissimilar but semantically alike locations on images. The two most frequent approaches to aligning two images are a homography matrix and a disparity map. In the case of a homography matrix, it is assumed that the transformation relationship between images is based solely on the planar areas of the ground or objects. However, a higher degree of freedom is necessary because the target to be aligned is all regions of images containing various forms of instances, which implies that there is a limit to matching all pixels with planar assumption alone. In contrast, a disparity map indicates the extent of misalignment between corresponding pixels in an image pair, allowing for robust matching regardless of the degree of freedom. Nonetheless, in a multispectral setting, it is difficult to obtain corresponding points using pixel intensity-based matching methods due to the visual representation difference between images from different sensors, which leads to inaccurate disparity. In this section, to solve the preceding problems, a disparity approximation scheme is introduced for robust alignment independent of degrees of freedom while matching corresponding pixels between multiple sensors more accurately.

In general, obtaining a disparity map between images captured by two RGB cameras begins with the elimination of sensor calibration-induced distortion. After that, the epipolar lines are calculated based on the epipole of the two images determined from extrinsic parameters of two sensors obtained during the sensor calibration procedure, and then the epipolar lines are rectified to lie horizontally to each other. As a result, the candidates for corresponding pixels are located in a horizontal area of the same height in the opposite image. Finally, the disparity map can be computed following a cost-matching operation that selects corresponding pixels from the candidate pool in the rectified images. In this way, cost matching after rectification is a commonly used order in calculating a disparity, and if this order is simply applied to the multispectral setup, it can be formulated as Equation (1). *v* and *t* refer to images acquired from RGB cameras and thermal cameras, undistorted and resized at the same resolution, respectively. $\omega_{v\cdot t}$ refers to transformation via a rectification matrix between the image pairs. $\phi \langle \cdot ,\cdot \rangle$ refers to a function that performs a cost matching given two inputs, and it leverages the widely used Semi-Global Matching (SGM) [22] method. $d_{v\to t}$ refers to a disparity map containing the extent of misalignment to overlap the corresponding points from *v* and *t*.

(1) $$d_{v\to t}=\phi \langle \omega_{v\cdot t}\left(v\right),\;\omega_{v\cdot t}\left(t\right)\rangle$$

However, as previously indicated, data acquired from different sensors in a multispectral setup depict the same scene differently in terms of pixel intensity, so utilizing the existing cost matching algorithm that assumes input images are all in the same RGB domain leads to a very imprecise output due to the intensity imbalance. This issue is circumvented by exploiting the disparity between RGB sensors estimated from our synchronized multispectral configuration. Specifically, the disparity map $d_{v(l)\to v(r)}$ between left RGB v(l) and right RGB v(r) images can be calculated because the inputs do not deviate from the assumption of the cost matching function, and in this case, $d_{v(l)\to v(r)}$ has the same resolution as v(l) and v(r). To approximate the disparity between the RGB camera and the thermal camera by converting $d_{v(l)\to v(r)}$, the inverse transformation of the rectification applied to v(l) is performed on $d_{v(l)\to v(r)}$ to generate a disparity of raw images. Consequently, the disparity $d_{v(l)\to t(l)}$ between different sensors is determined by applying the rectification operation between v(l) and t(l) (left thermal camera) to the inverse transformed $d_{v(l)\to v(r)}$. This procedure can be formulated as Equation (2), and two images from multi-modal sensors are aligned through pixel-level translation using the estimated disparity.

(2) $$\begin{array}{cc} d_{v(l)\to v(r)} & =\phi \langle \omega_{v(l)\cdot v(r)}\left(v(l)\right),\;\omega_{v(l)\cdot v(r)}\left(v(r)\right)\rangle \\ d_{v(l)\to t(l)} & =\omega_{v(l)\cdot t(l)}\left(\omega_{v(l)\cdot v(r)}^{-1}\left(d_{v(l)\to v(r)}\right)\right) \end{array}$$

#### 2.1.5. Annotation

All images, 19,420 per camera, were manually annotated with Computer Vision Toolbox [23] by highly experienced computer vision researchers using the following heuristic rules:

- Only images captured by a single RGB camera are annotated, as the rest of the images can be interpreted using the same annotation after being geometrically aligned using the direct linear transformation (DLT), which includes rotation and translation.
- To obtain more accurate centroid distance annotations, we modified the SGM hyperparameters and then employed a hole-filling method to reduce error. After manually removing outliers, we used the average depth value surrounding the centroid pixel of each pedestrian.
- For a more specific annotation class, we defined three class labels, each of which represents information about what the instance contains, as follows: (1) person; (2) people; (3) background.
- Occlusion information is also included for each annotated instance based on the degree of occlusion, which is defined, as follows: (1) no-occlusion (0%); (2) partially-occluded (0–50%); (3) strongly-occluded (50–100%), respectively. This information is crucial for real-world applications, as all circumstances must be taken into account to prevent a collision.

#### 2.1.6. Considerations Made during Dataset Collection Process

In the KAIST dataset, they used a braking distance range when cars have a typical driving speed, analyzed the corresponding height sizes in pixels when a pedestrian is approximately 1.7 m tall, and used the sizes to establish subcategories to determine performance differences based on the size of pedestrians. However, directly using the same criterion is not reasonable due to the distinguishing features of cars and forklifts. Specifically, forklifts are relatively slower than cars. According to industry standards and guidelines ANSI B56.1 [24], there is no such a strict regulation but it is recommended that the speed of indoor forklifts should not exceed 10 km/h, and even some companies limit the speed to 5 km/h [25]. Therefore, for better safety, we adopt the 5 km/h speed limit and define subcategories based on the braking distance at 5 km/h, as follows: (1) hazardous zone when the distance of pedestrians is closer than the braking distance; (2) warning zone when the distance of pedestrians is greater than the braking distance.

In order to calculate the braking distance at 5 km/h and the corresponding height size of pedestrians in pixels, we first analyzed the characteristics of forklifts. Forklifts are designed to transport heavy objects, such as containers and palettes. In other words, heavy payloads can have a substantial effect on the braking distance of forklifts, resulting in a different braking distance from that of cars. Consequently, the braking distance of forklifts can be calculated using the velocity, total weight including payloads, and the floor status [26]. However, the theoretical braking distance and the actual braking distance may differ for a variety of reasons, including inaccurate measurements, the shape of payloads, the drag force, etc. Therefore, we applied linear interpolation to statistical values presented in the article [26] to determine the actual braking distance at 5 km/h, and we believe this is reasonable because the statistical values nearly follow a linear distribution. As a result, the braking distance of forklifts operating on a flat, dry concrete surface or equivalent is approximately 2.2 m at 5 km/h, and the corresponding height size is about 320 pixels in our dataset domain. Consequently, we set 320 pixels as a threshold value when determining the subcategories we defined, i.e., hazardous zone and warning zone. Moreover, for autonomous vehicles, it is preferable to narrow down the candidate pool based on their sizes, as detecting too small pedestrians is redundant when braking distance is taken into account. Therefore, we set the height range of the “warning zone” to be between 55 and 320 pixels, and the height of the “hazardous zone” to be greater than 320 pixels.

In addition, the distance traveled while a driver responds to an emergency is approximately 4.17 m [26]. Ideally speaking, automated forklifts do not need human supervision, so the distance traveled while a driver responds to an emergency does not have to be considered. However, for automated forklifts that still need human supervision and for a safety margin, we determined the reasonable distance range to be from 0 meters to the sum of two distances 5.63 m + 4.17 m = 9.8 m. Therefore, when capturing images, the distance of pedestrians is mostly set between 0 and 10 m, which corresponds to the braking distances when the forklift is at a standstill and at its speed limit, including the human’s response time.

### 2.2. Proposed 2.5D Multispectral Pedestrian Detection Algorithm

**Figure 4:**
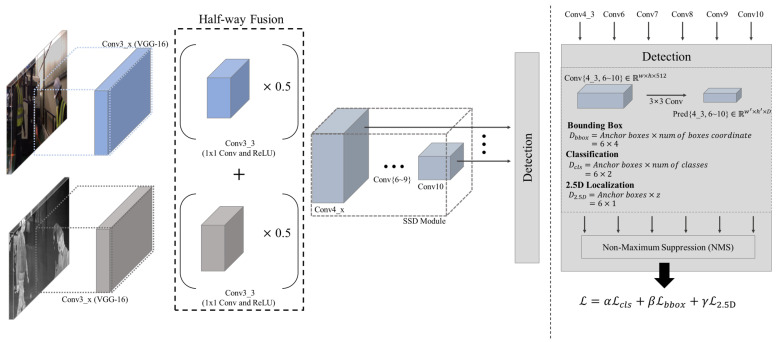
Architecture of the 2.5D detection model proposed. The proposed 2.5D detection model receives RGB and thermal images as inputs, fuses both features in middle convolution layers by adding two features after multiplying both features by a weight factor of 0.5, and employs six different fused features to perform pedestrian detection and centroid localization. In this manner, the proposed 2.5D detection model can exploit low-level and high-level features. Then the detection head processes the six feature maps independently before concatenating all predicted outputs along a channel axis for use in the final detection results.

SSD [27] is regarded as one of the most fundamental architectures for pedestrian detection among single-stage detectors. In SSD, default boxes and multi-scale features are used, and this strategy achieved remarkable performance in light of the trade-off between speed and accuracy. Since the advent of SSD, many recent works [2,28] have also used the main concepts that were introduced in SSD. Therefore, to make it more general, we developed 2D and 2.5D multispectral pedestrian detectors based on SSD for baseline performance and validated the presented dataset using these detectors. To achieve the objective, we first modified SSD to take multi-modal inputs. In particular, in contrast to single-modal models, multispectral detectors take RGB and thermal images as inputs to extract meaningful embedding features from them. Therefore, we design two independent CNN layers to extract features from both modalities and fuse them in middle convolution layers, as it is shown in Figure 4. For the fusion method, we employ the same technique called Halfway Fusion referring to previous studies [14,16,17] to combine features from both modalities. Specifically, the feature fusion process can be simply formulated, as follows:

(3) $$f_{m}^{i}=F(0.5f_{R}^{i}+0.5f_{T}^{i})$$

where *i* indicates the index of the layer i∈{4_3,6,7,8,9,10} in which the feature fusion occurs. In Equation (3), multi-modal features $f_{m}^{i}$ can be calculated by adding weighted RGB features $0.5f_{R}^{i}$ and thermal features $0.5f_{T}^{i}$. As a result, six multi-modal features $f_{m}^{i}$ that have the same resolution and channels as the input features from both modalities are obtained.

(4) $$Pred_{j}=Conv_{j}^{4\_3}\left(f_{m}^{4\_3}\right)⊕Conv_{j}^{6}\left(f_{m}^{6}\right)⊕\cdots ⊕Conv_{j}^{i}\left(f_{m}^{i}\right)⊕\cdots ⊕Conv_{j}^{10}\left(f_{m}^{10}\right)$$

where $f_{m}^{i}$ refers to multi modal features and j∈{bbox,cls,loc} indicates one of the following: (1) bounding box regression bbox; (2) classification cls; (3) 2.5D localization loc. The multi-modal features $f_{m}^{i}$ are fed into the detection head and independently pass through the *i*th convolution layer $Conv_{j}^{i}$ with a 3 × 3 kernel, padding size 1, and the different number of output channels depending on *j*. For example, $Conv_{bbox}^{i}$ has 6 × 4 output channels, which represent the estimated offsets in cartesian coordinates, i.e., x, y, w and h, for six anchor boxes. Similarly, $Conv_{cls}^{i}$ has 6 × 2 output channels, including background and person class for six anchor boxes, and the number of output channels of $Conv_{loc}^{i}$ is 6 × 1, which represents the centroid depth values for six anchor boxes. The six independent feature maps $Conv_{j}^{i}\left(f_{m}^{i}\right)$ are all concatenated in a channel axis with a concatenation operator ⊕. As a result, the aggregated feature map $Pred_{j}$ containing all default box information from low-level features to high-level features for *j* is obtained.

Then loss terms for bounding box regression, classification and 2.5D localization can be calculated, as follows:

(5) $$L_{bbox}=\frac{1}{N}\sum_{k=1}^{N}\left|Pred_{bbox}^{k}-g_{bbox}^{k}\right|$$

(6) $$L_{cls}=-\frac{1}{N}\sum_{k=1}^{N}\left(g_{cls}^{k}\cdot log\left(Pred_{cls}^{k}\right)+\left(1-g_{cls}^{k}\right)\cdot log\left(1-Pred_{cls}^{k}\right)\right)$$

(7) $$L_{loc}=\frac{1}{N}\sum_{k=1}^{N}\left|Pred_{loc}^{k}-g_{loc}^{k}\right|$$

where *k*, *N* and log indicate the index of the anchor boxes, the number of anchor boxes in $Pred_{j}$, and the natural logarithm, respectively. $g_{j}^{k}$ is the ground truth corresponding to $Pred_{j}^{k}$. Finally, the total loss term $L_{tot}$ can be simply calculated by the weighted summation of three loss terms, as follows:

(8) $$L_{tot}=\alpha L_{bbox}+\beta L_{cls}+\gamma L_{loc}$$

where α,β,γ are the weight factors of each loss term. In all experiments, α,β,γ are all set to 1.

## 3. Results

### 3.1. Proposed Multispectral Pedestrian Dataset

In order to determine the advantages of the proposed dataset, we compare the proposed dataset to previous benchmark datasets used in thermal pedestrian detection in Table 2. First, the LSI, KMU, CVC-14, and PTB-TIR datasets only collected thermal images, so texture information is insufficient due to the absence of an RGB image. In order to take advantage of RGB images, the KAIST dataset, which consists of pixel-level aligned multispectral image pairs, was proposed; the KAIST dataset has been used as a benchmark dataset for multispectral pedestrian detection. However, all of the aforementioned datasets were captured in a street domain for driving cars, making it challenging to apply them to other domains. In the meantime, the LLVIP dataset was proposed, which is a large-scale aligned pixel-level dataset for a visual surveillance domain. However, the LLVIP dataset is still not suitable for automated forklifts and the intralogistics domain due to the domain gap between indoor and outdoor applications. In addition to the limitations, the aforementioned datasets only include mono cameras in their camera sensor packs, whereas stereo cameras are more advantageous for stereo-vision research.

#### Distribution and Characteristics of Proposed Dataset

**Figure 5:**
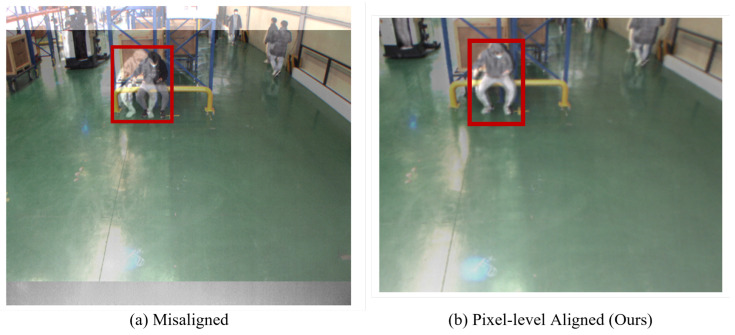
RGB and thermal images are overlaid to determine whether they are overlapping each other. The pedestrians in RGB and thermal images (Red box) are misaligned (**a**), but after applying the proposed method, they are pixel-level aligned (**b**).

To validate the alignment method applied to the proposed dataset, we visually compare the RGB-thermal image pairs before and after the alignment method is applied by overlaying RGB and thermal images. As a result, as shown in Figure 5a, RGB-thermal image pairs are not properly aligned, particularly for the pedestrian in the image. After applying the alignment method, however, it is demonstrated that the RGB-thermal image pair is pixel-level aligned (Figure 5b), making the proposed dataset more applicable to practical applications.

**Figure 6:**
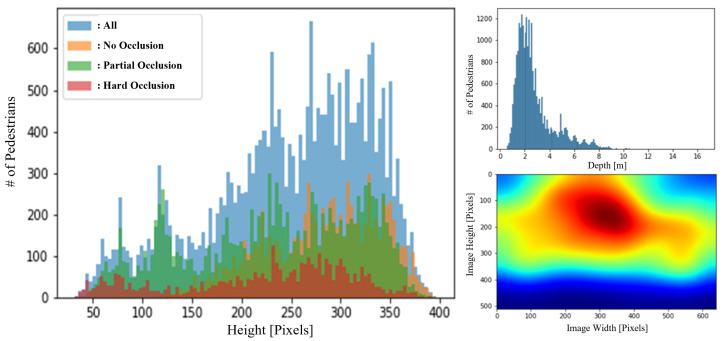
Distribution of the number of pedestrians over their height sizes in pixels depending on occlusion labels (**Left**). Distribution of the number of pedestrians depending on their centroid depth value (**Right Top**). Distribution of the centroid locations of pedestrians (**Right Bottom**). # refers to ’the number’ in the figure.

Occlusion is one of the most important issues that must be addressed in order to prevent an accident. For instance, if a warehouse worker is severely occluded by objects, automated forklifts may fail to detect the worker or estimate the distance accurately, resulting in a collision. Consequently, it is essential to know how many occluded pedestrians the dataset contains. For these reasons, we included occluded pedestrians in all size ranges, as shown in Figure 6.

**Figure 7:**
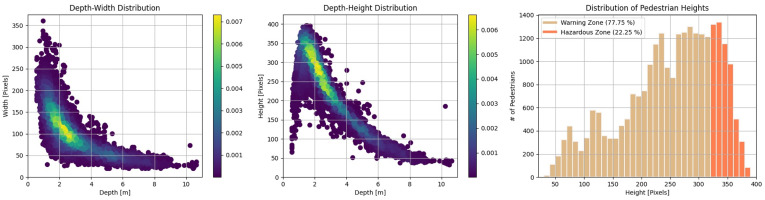
Relationships between depth and width (**Left**) and depth and height (**Middle**). Height distribution in the warning zone and hazardous zone (**Right**).

In order to design a depth regression model, we analyze the correlation between depth and the size of bounding boxes, i.e., width and height, as shown in Figure 7. According to MonoLoco [32], if the height of pedestrians is consistent, there should be no ambiguity, and their distribution is linear depending on age and gender groups. However, in real-world applications, the height of pedestrians cannot be a fixed value because of their individual identities. Therefore, it is worthwhile to analyze the relationship between the size of bounding boxes and depth to determine what types of features to employ for a depth regression model.

### 3.2. Results of Proposed 2.5D Multispectral Pedestrian Detection Algorithm

In this section, the outcomes and implications of the proposed 2.5D multispectral detection algorithm will be discussed. In contrast to conventional detection algorithms, the proposed algorithm includes a 2.5D localization branch that estimates the distance of detected pedestrians’ centroid w.r.t. the camera’s position in order to inform automated forklifts of the 3D location of warehouse workers.

Before discussing the results of the proposed 2.5D detection model, we would like to briefly discuss our first attempt to obtain the centroid distance in this section. In Section 3.1, it is demonstrated that centroid distance values correlate with the width and height of pedestrians. In addition, according to MonoLoco [32], the localization error caused by variations in human height at different camera distances follows nearly a linear distribution depending on age and gender groups. Therefore, the most naive approach is to directly regress the centroid distance from the given bounding box coordinates, so we first designed a centroid regression model consisting of fully connected (FC) layers with and without activation functions in order to compare the performance of centroid regression models with linear and non-linear representation capabilities, which we call naive centroid regressor (NCR).

Then, as described in Section 2.2, we designed a 2.5D localization model by adding an additional branch to the SSD 2D model to determine which method between the naive approach and the additional branch to SSD is more effective for 2.5D localization.

#### 3.2.1. Experiment Setups

The implementation is based on SSD in PyTorch with a 2.10 GHz Intel(R) Xeon(R) Gold 5218R CPU and an NVIDIA GeForce RTX 3090 GPU. As a backbone network for feature extraction, VGG16 pre-trained on ImageNet is used. The aspect ratio for anchor boxes are set to 1/1 and 1/2 with fine scales $\left[2^{0},2^{1/3},2^{2/3}\right]$, while scale levels are set to 40, 80, 160, 200, 280, and 360. The model is trained using stochastic gradient descent (SGD) with initial values for learning rate, momentum, and weight decay of 0.001, 0.5, and 0.0005, respectively. For the naive centroid distance regression, two FC layers followed by dropout and batchnorm are used. The first FC layer takes 4 inputs, namely x, y, w, and h, while the subsequent FC layer receives the 64 inputs and outputs a centroid depth value.

#### 3.2.2. Evaluation Metrics

To evaluate detection performance, average precision (AP) and log-average miss-rate (MR) [33] are adopted. These two metrics are commonly used for evaluating detection performance of multispectral pedestrian models [2,3,4]. Particularly, MR is utilized more frequently for pedestrian detection, where a low miss rate is essential for driving safety.

In addition, we employ percent error to evaluate the predicted centroid distance referring to [34].

(9) $$Percent\;Error=\frac{1}{N}\sum_{n=1}^{N}\left|\frac{Z_{n}-{\hat{Z}}_{n}}{Z_{n}}\right|\times 100\%$$

where *N* is the number of predicted bounding boxes. $Z_{n}$ and ${\hat{Z}}_{n}$ indicate the predicted and ground truth distance values between the centroid of *n*th pedestrian and camera position, respectively. We also adopted average localization precision (ALP) [32] for the same purpose. ALP considers a prediction to be correct if the error between the predicted centroid distance and the ground truth centroid distance is less than a threshold.

#### 3.2.3. Experimental Results

To verify the proposed 2.5D multispectral detection model, we first evaluate detection performance w.r.t. AP and MR depending on modalities, i.e., RGB, thermal, and multi, and dimensions, i.e., 2D and 2.5D. As it is shown in Table 3, the detection performance of the model is at its peak when the centroid regression branch is added and both RGB and thermal modalities are employed. The results indicate that the proposed 2.5D model can not only localize detected pedestrians in 3D space, but also maintains or even outperforms the detection performance of 2D models by exploiting both modalities.

**Figure 8:**
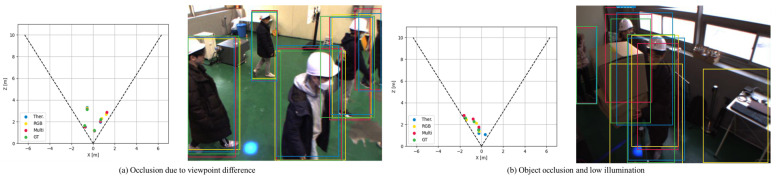
Qualitative results of 2D detection and 2.5D localization. (**a**) Detection and localization results depending on modalities when occlusion occurs due to viewpoint differences; (**b**) Detection and localization results depending on modalities under a low illumination condition.

Then, to validate the centroid regression performance of the proposed model, we evaluate the naive centroid regression model (NCR) and the centroid regression result from the proposed 2.5D detection model (SSD 2.5D) w.r.t. percent error and ALP. Consequently, it is demonstrated that NCR with activation functions outperforms NCR without activation functions, implying that the distribution between box information, namely x, y, w, and h, and the depth value is non-linear, due to age, gender, and height of pedestrians. Therefore, it is verified that NCR with activation functions that have a non-linear representation capability is more suitable for centroid localization. On the other hand, it is demonstrated that the centroid localization performance of the proposed SSD 2.5D model even outperforms NCR with activation functions for all modalities in terms of both percent error and ALP, as it is shown in Table 4 and Table 5. We believe this is due to the fact that the proposed SSD 2.5D model can account for semantic information when regressing the centroid depth value. The qualitative results of the detection and localization depending on modalities are shown in Figure 8. In conclusion, if you want to design a model that can simultaneously detect pedestrians and localize them in 3D space, i.e., centroid regression, it is more advantageous to design a model that can take semantic information around the detected pedestrian and regress the centroid distance given the surrounding features based on experimental results.

## 4. Conclusions

In this paper, we have discussed multispectral pedestrian detection to assign a human-aware characteristic to automated forklifts in order to prevent collisions in intralogistics. To achieve this, we first collect a multispectral pedestrian dataset in a real warehouse using four camera sensors mounted on a moving forklift. The dataset employs a method that aligns image pairs from different domains, i.e., RGB and thermal, without the use of a cumbersome device such as a beam splitter, instead utilizing the disparity between RGB sensors and camera geometry. In addition, we propose a multispectral pedestrian detector named SSD 2.5D that can not only detect pedestrians but also estimate the distance between an automated forklift and workers. In extensive experiments, the performance of detection and centroid localization is validated with respect to evaluation metrics used in a driving car domain but with distinct categories, such as hazardous zone and warning zone, to make it more applicable to the intralogistics domain. In conclusion, it is confirmed that the proposed 2.5D multispectral detection model SSD 2.5D provides the best detection performance in most cases, and outperforms all other comparisons in terms of localization performance.

## Figures and Tables

**Table 1:** Camera Configuration.

	RGB 1,2	Thermal 1	Thermal 2
Width	1288	320	320
Height	964	256	256
Sycn. Mode	Mode 14	Self Master	External Master
VCC	4	1	1
Ground	5	2	1
Sync. In	4(GPIO 3)	x	5
Sync. Out	x	3	3
Image Format	Raw8	Mono8	Mono8
Shutter Speed	15	x	x

**Table 2:** Comparisons between pedestrian datasets.

	Training		Testing		Properties								
	Peds	Images	Peds	Images	Frames	RGB	Ther.	Depth	Occ. Labels	Moving Cam	Vid. Seqs	Aligned	Domain
KAIST [8]	41.5 k	50.2 k	44.7 k	45.1 k	95.3 k	Mono	Mono	-	🗸	🗸	🗸	🗸	Streets
CVC-14 [9]	4.8 k	3.5 k	4.3 k	1.4 k	5.0 k	Mono	Mono	-	-	🗸	🗸	-	Streets
LSI [29]	10.2 k	6.2 k	5.9 k	9.1 k	15.2 k	-	Mono	-	🗸	🗸	🗸	-	Streets
KMU [30]	-	7.9 k	-	5.0 k	12.9 k	-	Mono	-	-	🗸	🗸	-	Streets
PTB-TIR [31]	-	-	-	-	30.1k	-	Mono	-	🗸	🗸	🗸	-	-
LLVIP [5]	33.6 k	12.0 k	7.9 k	3.5 k	15.5 k	Mono	Mono	-	-	-	🗸	🗸	Surveillance
FLIR-ADAS [10]	22.3 k	8.8 k	5.7 k	1.3 k	10.1 k	Mono	Mono	-	-	🗸	🗸	-	Streets
Ours	12.6 k	7.3 k	20.6 k	12.2 k	19.5 k	Stereo	Stereo	🗸	🗸	🗸	🗸	🗸	Intralogistics

**Table 3:** Detection performance of the proposed SSD 2.5D model w.r.t. AP and MR on the proposed dataset. Warn. and Hazard. refer to the warning zone and hazardous zone, respectively. The highest performance is highlighted and bolded.

Model	Modality	Detection Result [%]					
		AP (↑)	AP Warn.	AP Hazard.	MR (↓)	MR Warn.	MR Hazard.
SSD 2D	RGB	61.6	61.5	45.2	4.10	4.31	6.39
SSD 2D	Ther.	59.5	59.2	41.2	4.41	4.69	7.82
SSD 2D	Multi.	65.2	65.2	40.5	3.53	3.59	8.81
SSD 2.5D	RGB	60.4	60.4	43.3	3.79	3.93	**5.52**
SSD 2.5D	Ther.	58.1	58.1	41.4	4.76	5.0	7.71
SSD 2.5D (Ours)	Multi	**65.7**	**65.7**	**45.7**	**3.24**	**3.33**	7.83

**Table 4:** Percent Error of the proposed SSD 2.5D model in comparison with the naive centroid regression method w.r.t. percent error. w/o Act. and w Act. refer to without activation functions and with activation functions, respectively. The highest performance is highlighted and bolded.

Model	Modality	Percent Error [%] (↓)						
		Total	1 m	2 m	3 m	4 m	5 m	>5 m
NCR (w/o Act.)	GT	38.25	3.97	25.88	29.48	31.46	34.30	36.27
NCR (w Act.)	GT	17.06	1.23	7.63	11.17	12.27	14.18	15.56
SSD 2.5D	RGB	6.16	0.36	4.27	5.84	5.98	6.08	6.1
SSD 2.5D	Ther.	6.27	0.36	4.2	5.94	6.1	6.22	6.24
SSD 2.5D (Ours)	Multi	**4.09**	**0.22**	**2.86**	**3.71**	**3.84**	**3.96**	**4.02**

**Table 5:** Average Localization Precision (10%). The highest performance is highlighted and bolded.

Model	Modality	Average Localization Precision (↑)						
		Total	1 m	2 m	3 m	4 m	5 m	>5 m
NCR (w/o Act.)	GT	0.1587	0.0222	0.1406	0.2106	0.2001	0.1832	0.1712
NCR (w Act.)	GT	0.4348	0.0044	0.5103	0.5166	0.5103	0.4851	0.4616
SSD 2.5D	RGB	0.7958	0.5268	0.7151	0.7534	0.7709	0.7828	0.7930
SSD 2.5D	Ther.	0.7594	0.4675	0.6793	0.7196	0.7359	0.7453	0.7546
SSD 2.5D (Ours)	Multi	**0.8571**	**0.5446**	**0.7984**	**0.8383**	**0.8483**	**0.8552**	**0.8593**

## Data Availability

The data presented in this study are available on request from the corresponding author. The provided data can be only used for non-profit purposes.
